# The effect of combining HIV latency reversal with inhibition of phosphoinositide-3 kinases or B-cell lymphoma-2 on the HIV reservoir

**DOI:** 10.1371/journal.ppat.1013923

**Published:** 2026-01-29

**Authors:** Youry Kim, Jenny L. Anderson, Carolin Tumpach, Ajantha Solomon, Jesslyn Ong, Kiho Tanaka, Jennifer M. Zerbato, Tania Tan, Charis E. Teh, Daniel H. D. Gray, Philip Arandjelovic, James McMahon, Niamh Meagher, David Price, Marc Pellegrini, Michael Roche, Sharon R. Lewin

**Affiliations:** 1 Department of Infectious Diseases, The University of Melbourne at the Peter Doherty Institute of Infection and Immunity, Melbourne, Australia; 2 Walter and Eliza Hall Institute of Medical Research, Melbourne, Australia; 3 Department of Medical Biology, The University of Melbourne, Melbourne, Australia; 4 Division of Infectious Disease and Immune Defence, The Walter and Eliza Hall Institute of Medical Research, Melbourne, Australia; 5 Department of Infectious Diseases, The Alfred Hospital and Monash University, Melbourne, Australia; 6 Centre for Epidemiology & Biostatistics, Melbourne School of Population & Global Health, The University of Melbourne, Melbourne, Australia; 7 Victorian Infectious Diseases Service, Royal Melbourne Hospital at the Peter Doherty Institute for Infection and Immunity, Melbourne, Australia; National Institutes of Health-NIAID, UNITED STATES OF AMERICA

## Abstract

The persistence of latently infected CD4 + T-cells in people with HIV (PWH) on suppressive antiretroviral therapy (ART) is the major barrier to an HIV cure. We investigated the impact of two classes of pro-apoptotic drugs, phosphoinositide-3 kinases (PI3K) inhibitors or the B cell lymphoma 2 (Bcl-2) inhibitor venetoclax on depletion of latently infected CD4 + T-cells when administered *ex vivo* either alone or in combination with a latency reversing agents (LRA) to induce expression of pro-apoptotic viral proteins. We quantified cell death in three latently infected cell lines (J-Lat clones) and the parental cell line (Jurkat) using a live dead stain and flow cytometry. Using CD4 + T-cells isolated from blood from PWH on ART, we quantified intracellular HIV RNA, integrated HIV DNA and intact proviral DNA using quantitative PCR. In the Jat10.6 latently infected cell line, the combination of an LRA with either a PI3K inhibitor or venetoclax, compared to an LRA alone resulted in higher levels of cell death. Using CD4 + T-cells from PWH on ART, there was a significant decrease in HIV DNA following administration of wortmannin (a pan-PI3K inhibitor), venetoclax (a Bcl2 inhibitor) and JQ1 (an LRA) when administered alone. There was minimal additional effect on reservoir reduction following the addition of an LRA with a pro-apoptotic drug, compared to either an LRA or pro-apoptotic drug alone. However, when CD4 + T-cells from PWH on ART were treated with LRAs combined with a PI3K inhibitor, the fold increase in cell associated unspliced HIV RNA correlated with the decline in HIV DNA. Overall, reduction in the HIV reservoir by LRAs could be further enhanced in the presence of pro-apoptotic drugs, but the magnitude of the effect was modest, was dependent on the *in vitro* model used and for PI3K inhibitors, depended on the potency of latency reversal. These results are consistent with minimal additional efficacy in reservoir reduction when combining currently available LRAs and either PI3K inhibitors or venetoclax.

## Author summary

The persistence of long-lived HIV-infected cells in people with HIV (PWH) on antiretroviral therapy (ART) is a major obstacle to finding a cure. We hypothesised that clearance of infected cells could be enhanced by combining pro-apoptotic drugs with latency reversing agents (LRAs) which will increase expression of pro-apoptotic viral proteins. We combined a range of LRAs with two different classes of drugs that promote cell death, inhibitors of PI3K and inhibitors of B-cell lymphoma-2, and tested the effects alone and in combination on the HIV reservoir in latently infected cell lines and cells from PWH on ART. Compared to an LRA alone, the combination of an LRA with either a PI3K inhibitor or venetoclax resulted in higher levels of cell death in the J-Lat10.6 latently infected cell line. Using CD4 + T-cells from PWH on ART, both classes of pro-apoptotic drugs reduced the HIV reservoir when used alone. There was minimal additional effect on reservoir reduction following the addition of an LRA with a pro-apoptotic drug, compared to either an LRA or pro-apoptotic drug alone. Overall, reduction in the HIV reservoir by LRAs could be further enhanced only modestly in the presence of pro-apoptotic drugs, but the magnitude of the effect was dependent on the *in vitro* model used and for PI3K inhibitors, on the potency of latency reversal. These results are consistent with minimal additional efficacy in reservoir reduction when combining currently available LRAs and either PI3K inhibitors or venetoclax.

## Introduction

The persistence of long-lived and proliferating latently infected CD4 + T-cells in people with HIV (PWH) during ART is the main barrier to finding a cure for HIV [1,2]. Because latently infected cells express minimal viral antigen and cannot be distinguished from uninfected cells, one approach being pursued is to enhance HIV transcription by latency reversing agents (LRAs) [3]. Multiple clinical trials of LRAs, given either alone or in combination with strategies to enhance immune clearance, have not shown a significant reduction in infected cells [4–7] or an increase in time to viral rebound post cessation of ART [8–10]. One explanation for this is the upregulation of anti-apoptotic proteins in latently infected cells that will favour cell survival. We hypothesised that clearance of infected cells could be enhanced by combining pro-apoptotic drugs with LRAs which will increase expression of pro-apoptotic viral proteins.

Apoptosis is a non-inflammatory form of programmed cell death that clears damaged or unwanted cells [11]. There are two major pathways regulating apoptosis: the intrinsic (or mitochondrial) and extrinsic (or death receptor) pathways. The intrinsic pathway of apoptosis is tightly regulated by interactions among members of the B-cell lymphoma (Bcl)-2 family of proteins, which contain both pro-apoptotic and anti-apoptotic functions [12]. Bcl-2 is an anti-apoptotic protein that binds and sequesters pro-apoptotic proteins such as Bak and Bim [12]. Antagonists of Bcl-2 exert their inhibition by binding with higher affinity to Bcl-2 proteins than the pro-apoptotic proteins, resulting in cell death [13].

Venetoclax is a Bcl-2 inhibitor approved for the management of chronic lymphocytic leukemia and can sensitise malignant cells to apoptosis both *in vitro* [14] and *in vivo* [15]. We and other groups have shown that both productively and latently HIV-infected cells were sensitive to cell death following treatment with venetoclax [16–18]. When venetoclax was given to HIV-infected humanized mice on ART, there was a delay in viral rebound once ART was stopped [18]. In addition, venetoclax resulted in depletion of intact HIV DNA *ex vivo* in CD4 + T-cells from people with HIV on ART [18]. However, whether venetoclax depletes cells that expressed HIV proteins or transcripts, or truly latent cells remains unclear. Given that HIV proteins themselves can also directly impact apoptotic pathways, having both anti-apoptotic and/or pro-apoptotic functions [19], we hypothesised that death would be enhanced in cells that express HIV RNA or protein.

Phosphoinositide-3 kinases (PI3K) control a range of intracellular signalling pathways in leukocytes and are also critical for cell survival [20]. The family of PI3Ks can be divided into three different classes based on their activating signals, structure and substrate specificity [20]. Mammalian class I PI3Ks consist of four isoforms: PI3Kα, PI3Kβ, PI3Kδ and PI3Kγ that are heterodimers formed from a catalytic subunit (p110α, p110β, p110δ and p110γ respectively) and a regulatory unit [20,21]. The binding of Akt to the products of PI3K leads to activation of Akt, resulting in inhibition of apoptosis [22]. PI3K/Akt inhibitors have been shown to induce apoptosis in HIV infected macrophages and microglial cells [23,24]. The activity of Bcl-2 proteins can also be regulated by the PI3K family of proteins [24]. However, the effects of PI3K inhibitors – either pan or selective inhibitors - in HIV latently infected T-cell lines or cells from PWH on ART has never been evaluated.

HIV proteins such as Envelope, Vpu, Protease and Vpr can also promote apoptosis through a variety of mechanisms [25]. As an example, HIV Protease can cleave procaspase 8 to create a novel fragment termed Casp8p41, which independently induces apoptosis in productively infected T-cells [26,27]. The Bcl-2 protein can sequester Casp8p41 to inhibit the progression of apoptosis and addition of a Bcl-2 inhibitor such as venetoclax has been shown to release the peptide from Bcl-2, leading to apoptosis of the infected cell [28]. This suggests that combining latency reversal with Bcl-2 antagonism, or other inhibitors of cell survival, may enhance death of infected cells. Indeed, others have shown that when venetoclax was combined with T-cell activation using anti-CD3/CD28, there was a reduction in the frequency of infected T-cells *ex vivo* using CD4 + T-cells from PWH on ART [28]. Given that maximal T-cell stimulation is not a viable therapeutic pathway [29,30], other LRAs warrant evaluation in combination with Bcl-2 inhibitors or other pro-apoptotic drugs.

We therefore evaluated the impact of a panel of LRAs together with either inhibitors of PI3K or Bcl-2 in latently infected cell lines and CD4 + T-cells from PWH on ART to determine if an LRA together with a pro-apoptotic drug can enhance clearance of the HIV reservoir.

## Materials and methods

### Ethics statement

Use of samples was approved by Human Research Ethics Committees at the Alfred Hospital in Melbourne, Australia and the University of Melbourne, Australia. Clinical details for participants are described (S1 Table in S1 File). Written consent was provided by all participants.

### Study participants

People with HIV on suppressive ART for at least three years underwent leukapheresis as previously described [31]. In brief, all participants were male and median duration on ART was 25.8 years with a median CD4 count at enrolment of 645.5cells/ul.

### Compounds

The following compounds (summarised in S2 Table in S1 File) were dissolved in dimethyl sulfoxide (DMSO) to make stock concentrations stored at -80˚C: 10mM vorinostat (VOR; MK0683) (Selleck Chemicals, Houston, TX) 10mM panobinostat (PNB; LBH589) (Selleck Chemicals), 10mM romidepsin (RMD; FK228) (Selleck Chemicals), 10mM venetoclax (VNX; ABT-199) (Selleck Chemicals), 10mM wortmannin (WN; KY12420) (Sigma-Aldrich, St. Louis, MO), 1mM PMA (Sigma-Aldrich) and 10mM raltegravir (RGTV) (Selleck Chemicals). IPI-443 and IPI-3063 were provided by Infinity Pharmaceuticals (Cambridge, MA), reconstituted in DMSO at a stock concentration of 10mM, and stored at -20°C. The following compounds were reconstituted in DMSO and stored at -20˚C: 100μM bryostatin (BRY) (Sigma-Aldrich), 1mM JQ1 (Selleck Chemicals), 1mg/ml phytohaemagglutinin (PHA) (Sigma-Aldrich) and 1mM Ionomycin (IONO) (Sigma-Aldrich).

### Cell culture

J-Lat6.3, J-Lat10.6, J-Lat15.4 and Jurkat T-cells (NIH AIDS Reference Reagent Program) were cultured in RPMI-1640 plus 10% heat-inactivated fetal bovine serum (FBS) (Interpath, Vic, AUS), supplemented with 100U/ml penicillin (Life Technologies), 100µg/ml streptomycin (Life Technologies) and 29.2mg/ml L-glutamine (Life Technologies) (RF10). Human embryonic kidney (HEK) 293T cells (NIH AIDS Reference Reagent Program) were cultured in Dulbecco’s modified eagle medium (DMEM) supplemented with 10% FBS, 100U/ml penicillin, 100µg/ml streptomycin and 29.2mg/ml L-glutamine (DMEM10). All cells were maintained in a humidified 37˚C, 5% CO_2_ atmosphere.

### Latency reversal and cell death in cell lines

J-Lat6.3/10.6/15.4 T-cells were seeded at a concentration of 0.25x10^6^ cells/500μl in a 48 well plate. Three concentrations (100nM, 10nM and 1nM) of either IPI-443 or IPI-3063 and three concentrations of venetoclax (1000nM, 100nM and 5nM) were added to the cells and incubated for 24 hours at 37°C and 5% CO_2_. After 24 hours of PI3K inhibitor treatment, the LRAs: 1μM vorinostat, 30nM panobinostat, 40nM romidepsin, 12.5nM bryostatin or 16nM PMA + 0.5μM ionomycin were added and the cells were incubated for an additional 24 hours before cells were then harvested and stained with a Live/Dead Fixable Violet Cell Death Stain (Life Technologies) (as per manufacturer’s instructions). All samples (two replicates) were read on a BD LSR Fortessa using FACs DIVA software and analysed using FlowJo v10 (FlowJo, BD Biosciences, Franklin, NJ).

### Isolation of total and resting CD4 + T-cells from whole blood from healthy donors and people living with HIV

Peripheral blood mononuclear cells (PBMCs) were isolated from whole blood from healthy donors [32] or from people with HIV on suppressive ART for at least 3 years who underwent leukapheresis [31]. Ficoll-Paque (Amersham Pharmacia Biosciences AB, Sweden) density separation was used to isolate PBMCs. Total and resting CD4 + T-cells were then isolated from PBMCs using negative bead depletion as previously described [31,32] as well as using the EasySep Human CD4 + T-cell Isolation Kit (StemCell Technologies, Vancouver, Canada) as per manufacturer’s instructions.

### *Ex vivo* stimulation of total CD4 + T-cells

Total CD4 + T-cells were seeded at a concentration of 3-5x10^6^ cells/ml in RF10 + 1U IL-2 + 1μM raltegravir and treated with a combination of pro-apoptotic drugs and LRAs. 100nM IPI-443, IPI-3063 or wortmannin, or 5nM, 10nM or 100nM venetoclax (collectively termed pro-apoptotic compounds) were diluted in a background of RF10 + 1U IL-2 + 1μM raltegravir and added to the cells and incubated for 24 hours at 37°C and 5% CO_2_. Venetoclax was used at a maximum concentration of 100nM, consistent with clinically achievable plasma levels, and this concentration has been shown previously [18] to selectively induce apoptosis in CD4 + T-cells *ex vivo.* After 24 hours, 20nM romidepsin, 30nM panobinostat, 12.5nM bryostatin or 1μM JQ1 was added, in the background of the pro-apoptotic compounds. Four hours after the addition of romidepsin, cells were washed 3 times and then resuspended in media that contained pro-apoptotic compounds in some instances or completely washed of all drugs and incubated a further 20 hours. 24 hours after the addition of panobinostat, bryostatin or JQ1, cells were washed and resuspended as above and incubated a further 48 hours. Cell pellets were washed 3 times and stored as pellets (2x10^6^ cells/condition) at -80°C for DNA extraction using the AllPrep DNA/RNA Mini Kit or QIAamp Kit (QIAGEN, Hilden, Germany) as per manufacturer’s instructions. To measure viability of cells, 0.2x10^6^ cells were stained with Live/Dead Fixable Near-IR Cell Death Stain (Life Technologies) at a concentration of 1:200 based off the manufacturer’s protocol. Cells were washed twice before being fixed in 1% paraformaldehyde and stored at 4°C, prior to analysis on a BD LSR Fortessa flow cytometer (in a single replicate). For RNA, 3x10^6^ cells/condition were pelleted and washed in phosphate buffered saline (PBS), then each sample resuspended in 350μl of RLT buffer (RNeasy Kit, QIAGEN) containing 1% β-mercaptoethanol (βME) (Sigma-Aldrich). Samples were homogenised through a QIAShredder spin column (QIAGEN) before storing the lysate at -80°C, prior to DNA/RNA extraction per manufacturer’s protocol.

### qPCR quantification of HIV Integrated DNA and unspliced RNA

Stored DNA pellets were thawed and lysed in 50μl of lysis buffer (10nM Tris HCl pH 8.0, 1mM EDTA, 0.002% Triton-X/1% SDS in H_2_O, ddH_2_O and 0.8mg/ml Proteinase K) as previously described [31]. Integrated HIV DNA (using four technical replicates for each donor) was quantified using a nested qPCR as previously described [31,33,34]. Cell-associated unspliced (CA US) HIV RNA and multiply-spliced (MS) HIV RNA was quantified using a semi-nested, reverse transcription (RT)-qPCR as previously described [31,35] (four technical replicates was used for RNA). qPCR results were analysed on MxPro qPCR software (Agilent Technologies).

### Intact proviral DNA assay

Intact and defective HIV provirus was quantified using primers and probes for the intact proviral DNA Assay (IPDA) as previously described [36] but adapted to the QIAcuity digital PCR machine (QIAGEN) [37]. Briefly, either 500ng (HIV DNA) or 25ng (RPP30) of genomic DNA (per well) was added to a master mix of HIV primers and probes, which included the restriction enzyme *Xho I* (New England Biolabs, Ipswich, MA). To eliminate the possibility of false positive PCR result, HIV DNA was extracted from healthy PBMCs and used as an additional negative control for the assay. DNA extracted from J-Lat6.3 T-cells was used as a positive control. Intact proviral DNA copies were not corrected for DNA shearing to avoid bias as previously described [18].

### Cytometry Time of Flight (CyTOF)

Following treatment of cells with pro-apoptotic drugs and latency reversing agents, cells were incubated with cisplatin (Standard BioTools, California, USA) in a 1:1000 dilution to a final concentration of 25μM for exactly 1 minute to discriminate dying cells. Four volumes of media containing serum was added to quench the reaction. Cells were then stained with IdU (1mM final concentration) and then fixed in 16% paraformaldehyde (Life Technologies) and stored in PBS containing 0.5% BSA. Fixed cells were barcoded with combinations of Palladium isotopes and stained as previously described [38] with a panel of antibody conjugates recognising the following intracellular and extracellular moieties: CD45 (85Y), HLA-DR (115In), CCR6 (141PR), CD19 (142Nd), CD45RA (143Nd), CCR5 (144Nd), CD8 (146Nd), ICOS (151Eu), CXCR3 (156Gd), CCR4 (158Gd), CCR7 (159Tb), CD69 (162Dy), CD45R0 (165Ho), CD27 (167Er), CD25 (169Tm), CD3 (170Er), CXCR5 (171Yb), CD38 (172Yb), CD4 (174Yb), PD-1 (175Lu), CD127 (176Yb), BAK-7D10 (140Ce), Bcl-XL (153Eu), BAX-1B4 (154Sm), Bcl-2 (157Gd), Mcl-1 (160Gd), Iκβα (164Dy), Ki67 (168Er) and cCaspase3 (173Yb). A commercial kit (MaxPar Human T-cell Phenotyping Panel Kit (Standard BioTools) containing T-cell subset markers were used in these experiments (Standard BioTools). Samples were run on a CyTOF-Helios (Standard BioTools) and analysed using Cytobank Software.

### Bliss Independence Model of synergy

The Bliss Independence Model for synergy was used to calculate the effects on the decline of integrated HIV between the pro-apoptotic drug and LRAs previously described [39]. If the two combinations of drugs have a Bliss Independence score greater than zero, then the combination was considered synergistic. A Bliss Independence score less than zero indicates that the combination of the two drugs display antagonism. Samples were calculated relative to the negative DMSO control. For the positive control, calculations were performed with the assumption of a 50% decline in integrated DNA.

### Data analysis and statistics

Graphs depicting the mean and errors bars showing standard error of means and graphs with means and 95% confidence intervals were generated on GraphPad Prism v9 (GraphPad Software, San Diego, USA). For statistical analysis, a paired T-test was used to determine statistical significance. Statistical analysis was performed on the data described in each figure. P-values less than 0.05 (p < 0.05) were considered statistically significant. Schematic diagrams throughout the manuscript were created using BioRender.com.

## Results

### Pro-apoptotic drugs together with LRAs significantly increase cell death or reactivation in J-Lat10.6 T-cells

**Fig 1:**
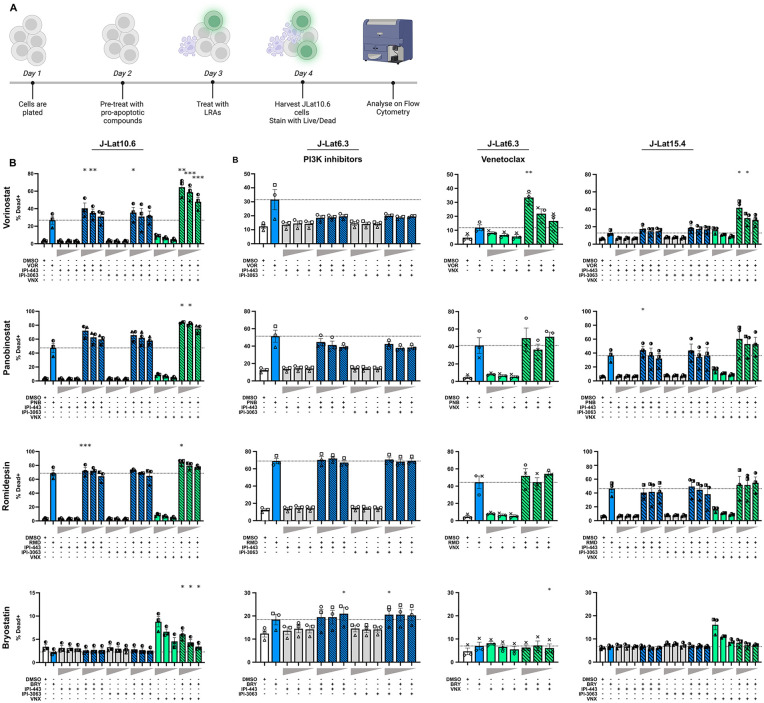
Histone deacetylase inhibitors combined with pro-apoptotic drugs results in higher levels of cell death compared to each LRA alone. **(A)** Schematic of the experimental plan. J-Lat T-cells were pre-treated with decreasing concentrations (grey horizontal triangles in panel B) of the PI3K inhibitors IPI-443 or IPI-3063 (100, 10 or 1nM) or venetoclax (1000, 100 or 5nM) for 24 hours. The latency reversing agents (LRAs) vorinostat (VOR; 1000nM), romidepsin (RMD; 40nM) panobinostat (PNB; 30nM) or bryostatin (BRY; 12.5nM) were then added to cell cultures for an additional 24 hours. Cells were harvested 24 hours after the addition of LRAs, stained with a Live/Dead Fixable Violet Dead Cell Stain, and analysed using flow cytometry. **(B)** The percentage of dead cells following stimulation with either: each LRA alone (blue), PI3K inhibitor alone (light grey) or in combination with each LRA (blue lined), Bcl-2 inhibitor alone (green) or combined with each LRA (green lined), is shown. Each symbol denotes an average of duplicate wells. Each experiment was performed three times, and the height of the column indicates the mean of the three independent experiments, with error bars showing the standard error of mean. The horizontal dashed line indicates the percentage of dead cells following treatment of J-Lat cells with the specific LRA alone. Experiments with J-Lat6.3 were conducted on two separate occasions, once with the PI3Ki and another with venetoclax. p-values were calculated using a paired t-test compared to the LRA treatment alone. *p < 0.05, **p < 0.01, ***p < 0.001. Created in BioRender. Kim, Y. (2026) https://BioRender.com/o9vg71a.

To investigate whether the combination of pro-apoptotic drugs together with LRAs to drive the expression of pro-apoptotic viral proteins would lead to an increase in cell death, J-Lat10.6 T-cells were treated for 24 hours with either PI3K inhibitors IPI-443 or IPI-3063 or Bcl-2 inhibitor venetoclax to first sensitize the cells to apoptosis. The LRAs vorinostat, panobinostat, romidepsin or bryostatin were then added to cultures to reactivate latent HIV and green fluorescent protein (GFP) was quantified (Figs 1A and S1A Fig in S1 File).

All LRAs induced expression of GFP reporter (S1B Fig in S1 File), as expected in J-Lat10.6 cell clones [40,41] while neither IPI-443, IPI-3063 nor venetoclax alone increased GFP expression, consistent with these pro-apoptotic compounds lacking LRA activity (S1B Fig in S1 File). The LRAs alone increased cell death, with romidepsin having the greatest impact on cell death (mean percentage of dead cells 68.62%), followed by panobinostat (47.41%) and vorinostat (26.85%). Bryostatin had minimal effect on cell death (2.27%), while the PI3K inhibitors had no effect on cell death on their own. Cell death was significantly increased with the combination of vorinostat with 100nM IPI-443, 10nM IPI-443 or 100nM IPI-3063 compared to vorinostat alone (p = 0.029, 0.005 and 0.030 respectively), and romidepsin with 100nM IPI-443 compared to romidepsin alone (p = 0.0003) (Fig 1B). While venetoclax alone had modest impact on cell death, cell death was significantly increased with high concentrations of venetoclax and all HDACi (Fig 1B–1D) demonstrating that the HDACi sensitised cells to venetoclax-induced cell death.

We next sought to confirm these findings in two other latently infected cell lines, J-Lat6.3 and J-Lat15.4 (Fig 1B), which are known to have different sites of HIV integration and therefore different responsiveness to latency reversal [42]. We observed minimal reactivation of virus for all LRAs in both cell lines (<1.5% of cells expressing GFP) except for romidepsin which increased GFP in all cell lines, but at different levels (mean GFP expression J-Lat6.3, 10.52%; J-Lat15.4, 3.51%; J-Lat10.6, 36.52%). In the J-Lat cell lines 6.3 and 15.4, none of the LRA and pro-apoptotic drug combinations significantly increased cell death versus LRA alone, apart from vorinostat combined with venetoclax (Fig 1B). However, given the low potency of latency reversal by vorinostat in CD4 + T-cells from PWH, we chose not to pursue this combination further [43]. The different findings in these two J-Lat clones compared to findings in J-Lat10.6 using romidepsin and venetoclax together, suggest that integration site could determine the response to an LRA but may also play a role in determining the likelihood of cell death.

To determine whether HIV integration and viral expression influenced the susceptibility to cell death from either LRAs or pro-apoptotic drugs in latently infected cell lines, we compared the effects of these drugs in parental Jurkat T-cells and the J-Lat10.6 T-cell line. Interestingly, the percentage cell death in the J-Lat10.6 T-cell line compared to the Jurkat T-cell line was lower following treatment with vorinostat alone (mean death 17.75% and 20.6% respectively), panobinostat alone (27.75% and 45.00%) and romidepsin alone (26.25% and 48.70%) (S2 Fig in S1 File). The pro-apoptotic drugs wortmannin and venetoclax alone (at 5nM, 10nM and 100nM) resulted in low levels of cell death in both cell lines (S1B Fig in S1 File). Similar to findings with the drugs alone, there were higher levels of cell death in the Jurkat T-cell line compared to J-Lat10.6 T-cell line for panobinostat and romidepsin together with venetoclax (S1C Fig in S1 File). These data are consistent with viral proteins not contributing to cell death in these cell lines.

Taken together, we demonstrated increased cell death using multiple HDACi and two classes of pro-apoptotic compounds in J-Lat10.6 cells. However, there was no significant change in cell death in J-Lat6.3 and J-Lat15.4 T-cells, where lower levels of GFP expression were observed. Given that latently infected cell lines are immortalised and therefore have dysregulated cell death pathways [44,45], we next investigated the effects of pro-apoptotic compounds in a more relevant model, primary CD4 + T-cells from PWH on ART *ex vivo*.

### Multiple LRAs combined with PI3K inhibitors lead to a decline in integrated HIV DNA

We assessed the impact of the pro-apoptotic drugs alone or in combination with either panobinostat, romidepsin, bryostatin or JQ1 in CD4 + T-cells from PWH on ART *ex vivo* (S2 Table in S1 File). Vorinostat was not tested in primary T-cells, given it has lower potency as an LRA *ex vivo* [35]. For pro-apoptotic drugs, we tested the Bcl-2 inhibitor venetoclax and three PI3K inhibitors: the selective IPI-443 and IPI-3063, plus a pan PI3K inhibitor, wortmannin [46].

Potential toxicity of the drug combinations was first examined on CD4 + T-cells from healthy donors. The timing of addition of LRA versus pro-apoptotic drug had little impact upon the toxicity of the drug combinations (S3A–S3C Fig in S1 File). Thus, we assessed the synergistic activity of the different compounds by adding pro-apoptotic drugs first to pre-sensitize cells towards apoptosis, followed by the addition of LRA to drive the expression of pro-apoptotic viral proteins. All drugs were administered for 24 hours (S3A–S3C Fig in S1 File), except romidepsin, which was incubated at 20nM for 4 hours, then washed out, to reduce toxicity to 35.58% average cell death (S3B Fig in S1 File).

**Fig 2:**
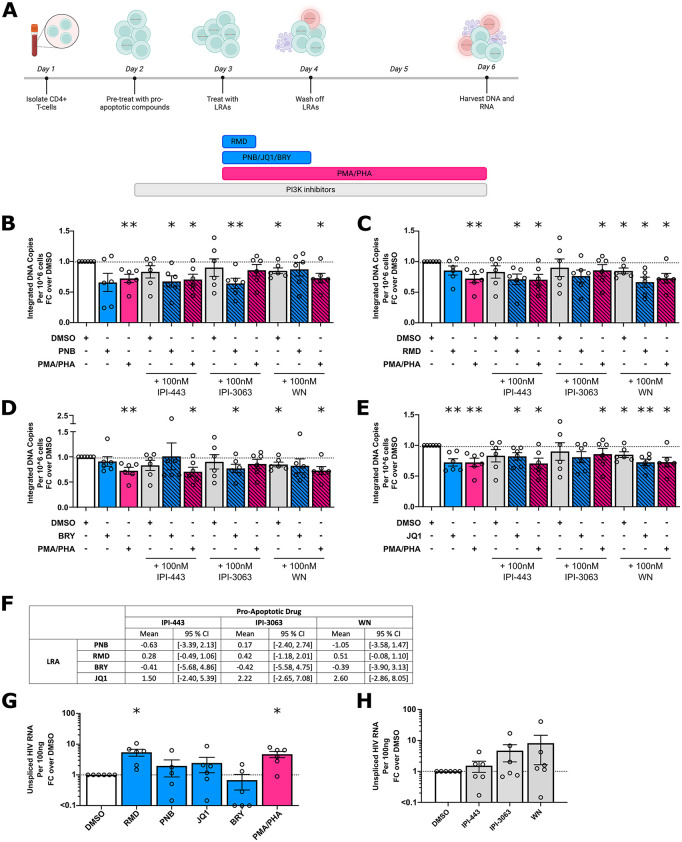
Histone deacetylase inhibitors or JQ1 with PI3K inhibitors compared to either drug alone induced a greater reduction in the levels of integrated HIV DNA in total CD4 + T-cells from PWH on ART *ex vivo.* **(A)** Total CD4 + T-cells were isolated from PBMCs collected from PWH on ART. Cells were then treated with pro-apoptotic compound [100nM IPI-443, 100nM IPI-3063 or 100nM wortmannin (WN)], or DMSO for 24 hours with and without an LRA. After incubation with the LRA, cells were washed twice in media and cultured for an additional 48 hours in media together with the pro-apoptotic drug. All samples were harvested and analysed for HIV integrated DNA per 10^6^ cells using qPCR for HIV integrated DNA and the house keeping gene CCR5. The fold change (FC) in integrated HIV DNA relative to DMSO (horizontal dashed line) is shown for each LRA including **(B)** panobinostat (PNB; 24-hour pulse of 30nM), **(C)** romidepsin (RMD; 4-hour pulse at 20nM); **(D)** bryostatin (BRY; 24-hour pulse of 12.5nM) or **(E)** JQ1 (24-hour pulse of 1μM). p-values were calculated using a paired t-test on fold-change data compared to DMSO, *p < 0.05, **p < 0.01. **(F)** The Bliss Independence Model was used to score each combination for the presence of synergy. A number > 0 is consistent with synergy; < 0 no synergy. Fold change in unspliced HIV RNA compared to DMSO is shown for **(G)** LRAs and **(H)** pro-apoptotic drugs. p-values were calculated using a paired t-test compared to DMSO, *p < 0.05. Each symbol represents a different donor, n ≤ 6. Columns represent the mean with error bars denoting standard error of the mean. Each bar graph for DMSO, LRA alone, pro-apoptotic drug alone or in combination with PMA/PHA shows the mean from different experiments to account for assay variation. Created in BioRender. Kim, Y. (2026) https://BioRender.com/2f2gii9.

The impact of the different LRAs together with pro-apoptotic PI3K inhibitors was tested on CD4 + T-cells from PWH *ex vivo*. The decrease in infected cells was measured by changes in integrated HIV DNA (Fig 2A–2F). For each drug alone compared to DMSO, WN at 100nM (mean fold change, MFC = 0.848, p = 0.023, Fig 2B–2D) and JQ1 (MFC = 0.723, p = 0.006, Fig 2E) resulted in a significant decline in integrated HIV DNA. Of note, there was no significant reduction in integrated HIV DNA following the selective IPI-443 and IPI-3063 PI3K inhibitors nor panobinostat, romidepsin and bryostatin when tested alone (Fig 2B–2D).

We next assessed combinations of drugs compared to DMSO and found a significant decline in integrated HIV DNA when panobinostat was combined with either IPI-443 (mean fold change, MFC = 0.677, p = 0.017) or IPI-3063 (MFC = 0.642, p = 0.008; Fig 2B); when romidepsin was combined with IPI-443 or wortmannin (MFC = 0.711, p = 0.021 and MFC = 0.663, p = 0.010 respectively; Fig 2C); and when the PKC agonist bryostatin was combined with IPI-3063 (MFC = 0.769, p = 0.040, Fig 2D), while neither panobinostat, romidepsin or bryostatin alone resulted in a significant decline in HIV DNA (Fig 2B–2D). JQ1 in combination with IPI-443 or wortmannin significantly decreased integrated HIV DNA compared to the DMSO control (MFC = 0.821, p = 0.032 and MFC = 0.723, p = 0.003 respectively; Fig 2E), as did JQ1 alone (Fig 2E). In a post-hoc analysis, we found there was no statistically significant difference in the decline in HIV DNA when we compared treatment with any LRA alone and the same LRA plus a pro-apoptotic drug*.*

To determine whether the combinations tested had a synergistic effect on the decline of infected cells, we applied the Bliss Independence Model (Fig 2F). We observed that when romidepsin or JQ1 were combined with any of the PI3K inhibitors, or when panobinostat was combined with IPI-3063, synergy was observed. However, there was no synergy with bryostatin and all three PI3K inhibitors, and panobinostat with IPI-443 or wortmannin. It’s important to note the wide confidence intervals in these calculations and the underlying assumption for the Bliss Independence calculation being that the pathways of the two drug treatments are mutually non-exclusive [47]. Thus, some caution is needed in interpretation.

Cell-associated unspliced (US) HIV RNA was also measured in the CD4 + T-cells from PWH on ART *ex vivo* treated with each drug alone to determine whether the LRAs or pro-apoptotic drugs alone reactivated latent HIV (Fig 2G). All LRAs reversed latency in the majority of donors with the exception of bryostatin (MFC in cell associated unspliced HIV RNA versus DMSO: romidepsin = 5.436; panobinostat = 1.969; bryostatin = 0.680; JQ1 = 2.452; PMA/PHA = 4.710; Fig 2G). These changes were significantly different from DMSO for romidepsin (p = 0.023) and the positive control PMA/PHA (p = 0.017) (Fig 2G). The PI3K inhibitors did not induce a significant increase in unspliced HIV RNA versus DMSO control (MFC IPI-443 = 1.535, IPI-3063 = 4.680 and WN = 8.189, p = 0.414, p = 0.223 and p = 0.322, respectively, Fig 2H). Although there was an increase in the mean CA US HIV RNA following each PI3K inhibitor compared to DMSO with the greatest changes seen with wortmannin, these changes did not reach statistical significance.

### Venetoclax alone or combined with romidepsin results in decreased integrated and intact HIV DNA

Previous research by our lab and others have shown that 100nM venetoclax alone selectively depleted HIV infected T-cells from PWH on ART *ex vivo* [18]. However, given the enhanced depletion of infected T-cells from PWH on ART with romidepsin and two of the PI3K inhibitors (Fig 2C), and latently infected J-Lat10.6 cells with romidepsin and venetoclax (Fig 1B), we next assessed the impact of romidepsin (as a representative potent HDACi) with venetoclax on death of HIV infected cells from PWH on ART *ex vivo*.

**Fig 3:**
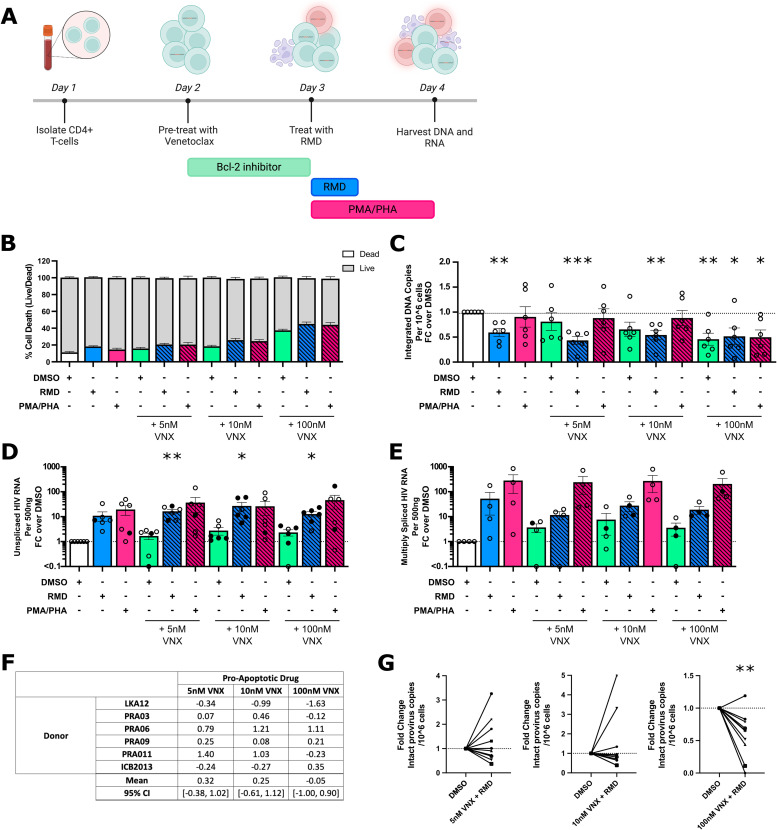
Romidepsin had no additional effect on the dose dependent decline in HIV DNA induced by venetoclax. **(A)** Total CD4 + T-cells were isolated from PBMCs collected from PWH on ART. Cells were then treated with venetoclax at three different doses: 5nM, 10nM and 100nM or DMSO for 24 hours. Cells were then either pulsed with 20nM romidepsin for 4 hours, washed of all drugs and cultured for an additional 20 hours; or cultured with PMA/PHA for 24 hours. All samples were harvested for DNA and RNA. **(B)** Toxicity of the drug treatments were measured using Live/Dead Near IR Cell Death staining and flow cytometry. The fold change (FC) in integrated HIV DNA relative to DMSO **(C)** is shown. Fold change compared to DMSO of: **(D)** unspliced HIV RNA per 500ng cellular RNA; and **(E)** multiply spliced HIV RNA per 500ng cellular RNA. The corresponding Bliss Independence Score **(F)** for the decrease in integrated HIV DNA is shown. Closed symbols had > 50ng but <100ng input RNA in the RT-qPCR, and the values have been adjusted for the equivalent of 100ng RNA input. **(G)** Fold change compared to DMSO of intact proviral DNA measured using the Intact Proviral DNA Assay (IPDA). One donor did not have detectable intact proviral DNA. Columns represent the mean of values and error bars standard error of mean. Each symbol represents a different donor, n ≤ 6. p-values were calculated using a paired t-test on fold change data compared to DMSO, *p < 0.05, **p < 0.01, ***p < 0.001. Created in BioRender. Kim, Y. (2026) https://BioRender.com/czfy1gn.

When CD4 + T-cells from PWH on ART *ex vivo* were treated with 5, 10 and 100nM venetoclax for 24 hours followed by a 4-hour pulse of romidepsin (Fig 3A), the mean percentage death was 15.88%, 18.4% and 37.42% of total cells respectively (Fig 3B). Consistent with our previous study [18], when cells were treated with the highest concentration of 100nM venetoclax alone, significant reductions in integrated HIV DNA were observed compared to the DMSO control (MFC = 0.458, p = 0.006) (Fig 3C). As expected, treatment of cells with romidepsin alone increased cell-associated unspliced (Fig 3D) and multiply spliced HIV RNA (Fig 3E) confirming latency reversal had been induced.

When romidepsin was combined with venetoclax and compared to DMSO, there was a statistically significant decrease in integrated HIV DNA with all three concentrations of venetoclax (5nM, 10nM and 100nM: MFC = 0.434, 0.543, and 0.516, p < 0.001, p = 0.004 and p = 0.033 respectively; Fig 3C). However, in a post-hoc analysis, when we compared venetoclax alone to venetoclax with romidepsin, there were no significant differences in the decline in HIV DNA (venetoclax concentration of 5nM: p = 0.517, 10nM: p = 0.907, 100nM: p = 0.698).This decline was also not synergistic (mean Bliss Independence scores of 0.32 (95% CI [-0.38, 1.02]) and 0.25 (95% CI [-0.61, 1.12]) respectively; Fig 3F). These data suggest that the addition of romidepsin to venetoclax at all doses, did not significantly increase a decline in integrated HIV DNA *ex vivo*.

Finally, given that quantification of integrated HIV DNA includes both intact and defective provirus, we also quantified the effect of romidepsin and venetoclax on intact proviruses in a larger set of donors using a modified Intact Proviral DNA assay (IPDA) [37]. We observed a significant decline in intact provirus in donors treated with 100nM venetoclax and romidepsin (MFC = 0.589, p = 0.005) compared to DMSO (Fig 3G). A statistically significant decline in intact HIV DNA was not observed with 5nM or 10nM venetoclax combined with romidepsin. When we compared romidepsin and venetoclax to venetoclax alone at the same concentration, there was no significantly significant decline in intact proviral DNA (5nM: p = 0.929, 10nM: p = 0.755, 100nM: p = 0.831). Finally, we observed a decrease in 5’ defective virus in 6/11 donors treated with 10nM venetoclax and romidepsin but little change in 3’ defective virus (Sf Fig in S1 File). Together these data demonstrate that cells with intact HIV DNA decreased in the presence of both romidepsin and venetoclax, but this was not greater than venetoclax alone. Interestingly, cells infected with 3’ defective virus were relatively resistant to depletion by this combination of drugs.

### Relationship between potency of latency reversal and cell death

**Fig 4:**
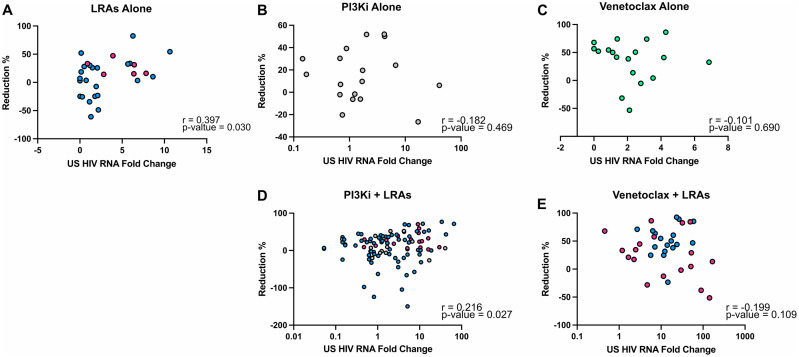
Positive correlation between the reduction of integrated HIV DNA and increase in unspliced HIV RNA in CD4 + T-cells from PWH on ART *ex vivo* treated with PI3Ki and LRAs. The percentage reduction in integrated HIV DNA compared to fold change in unspliced HIV RNA relative to DMSO was graphed after treatment of CD4 + T-cells from PWH on ART *ex vivo* with: **A)** LRAs alone (romidepsin, panobinostat, JQ1, bryostatin and PMA/PHA), **B)** PI3Ki alone, **C)** venetoclax alone, **D)** PI3Ki and LRAs, and **E)** venetoclax and LRAs. Correlations were calculated using a Pearson correlation coefficient. Each symbol denotes a separate donor (n = 6). Blue – LRAs: romidepsin, panobinostat, bryostatin, JQ1 alone or in combination with PI3Ki; pink – PMA/PHA alone or in combination with PI3Ki; grey – PI3Ki: IPI-443, IPI-3063, wortmannin; green – venetoclax.

To determine the mechanism of interaction between LRAs and pro-apoptotic drugs, we assessed the relationship between fold increase in HIV RNA and fold reduction in integrated HIV DNA for all combinations to determine if the reduction of HIV DNA was related to potency of HIV transcription induction. We first assessed latency reversing agents alone and showed a significant positive correlation between the fold increase in cell-associated unspliced HIV RNA and the reduction in the levels of integrated HIV DNA (r = 0.397, p = 0.030) (Fig 4A). When PI3Ki were combined with LRAs, the positive correlation between fold increase of HIV RNA and fold reduction of HIV DNA was again observed (r = 0.216, p = 0.027) (Fig 4B). However, this relationship was no longer observed with either venetoclax alone (r = -0.101, p = 0.690) (Fig 4C) or with venetoclax in combination with romidepsin (r = -0.199, p = 0.109) (Fig 4D).

These findings suggest that factors driving the decline in HIV DNA are different for PI3K inhibitors and venetoclax. It is possible that the HIV DNA decline for the combination of romidepsin and low dose venetoclax may be a consequence of the effects of both drugs on cell survival or cell death pathways active in HIV-infected cells, rather than a consequence of the induction of HIV RNA or protein [18,48].

### Venetoclax combined with romidepsin does not significantly change the levels of pro-apoptotic proteins

Given that there was no relationship between the fold increase in HIV RNA and decline in HIV DNA for the combination of romidepsin and venetoclax, we performed Cytometry time-of-flight (CyTOF) to measure a panel of apoptotic and CD4 + T-cell differentiation protein markers. We hypothesised that the decline in HIV DNA following the combination of romidepsin and venetoclax was a consequence of the impact of romidepsin on cell death pathways [48], rather than its effects on HIV transcription. We analysed CD4 + T-cells from PWH on ART *ex vivo* treated with different drug concentrations, PMBCs from uninfected donors, and two human myeloma control cell lines (KMS-12-PE (high Bcl-2 expression) and H9291 (low Bcl-2 expression)) (S6A and 6C Fig in S1 File).

In CD4 + T-cells from PWH on ART *ex vivo*, we observed a modest increase in the expression of Bak, Bcl-XL and cleaved caspase-3 (S6C and 6D Fig in S1 File) with the combination of venetoclax (10nM) and romidepsin compared to venetoclax (10nM) alone (MFC = 1.364, 1.397 and 1.220 respectively), or compared to DMSO control cells (MFC = 1.360, 1.443 and 1.373 respectively). Similar changes were also seen with an increase in Bcl-XL and caspase 3 (S5C and 5D Fig in S1 File) with venetoclax (100nM) and romidepsin compared to venetoclax (100nM) alone (MFC = 1.236 and 1.198, respectively) or DMSO control (MFC = 1.357 and 1.553, respectively).

## Discussion

We assessed whether death of infected cells could be enhanced in the presence of two classes of pro-apoptotic drugs either alone or when combined with an LRA. Compared to an LRA alone, the combination of an LRA with either a PI3K inhibitor or venetoclax resulted in higher levels of cell death in the latently infected cells line J-Lat10.6. Using CD4 + T-cells from PWH on ART, we showed a significant decrease in HIV DNA following administration of wortmannin (a pan-PI3K inhibitor), venetoclax (a Bcl-2 inhibitor) and JQ1 (an LRA) when administered alone. However, there was no difference in the decline in HIV DNA when we compared LRAs alone to LRAs with PI3K inhibitors. Similarly, there was no difference in the decline of HIV DNA or intact proviral DNA when cells were treated with venetoclax alone compared to a combination of venetoclax with romidepsin. When CD4 + T-cells from PWH on ART were treated with LRAs combined with a PI3K inhibitor, the fold increase in unspliced HIV RNA correlated with the decline in HIV DNA. Overall, reduction in the HIV reservoir by LRAs could be modestly enhanced in the presence of pro-apoptotic drugs, but the magnitude of the effect was small, was dependent on the *in vitro* model used and for PI3K inhibitors, was associated with the potency of latency reversal.

Currently, there are four FDA approved PI3K inhibitors for use in several types of cancer with many more in development. These include the PI3Kδ inhibitor Idelalisib for treatment of chronic lymphocytic leukemia [49] and follicular B-cell non-Hodgkin lymphoma, the dual PI3Kδ/γ inhibitor Duvelisib (IPI-145) for the treatment of chronic lymphocytic leukemia and small lymphocytic leukemia [50], a pan-PI3K inhibitor Copanlisib [51] for the treatment of follicular lymphoma, and the newly licenced PI3Kα inhibitor Alpelisib for the treatment of breast cancer [52]. We are unaware of any other prior work assessing the effects of PI3K inhibitors alone on depletion of the HIV reservoir. Recent work on a dual HDACi/PI3Ki fimepinostat (CUDC-907) demonstrated reactivation of HIV transcription in both T-cell line models and CD4 + T-cells from PWH on ART *ex vivo*, in the absence of T-cell proliferation or activation [53]. However, the effects of fimepinostat on cell death or HIV DNA levels was not explored. Other groups have demonstrated that PI3K inhibitors can reduce the potency of latency reactivation by HDAC inhibitors [54,55], which we did not observe in our study. In contrast, we found a greater reduction in integrated HIV DNA following the administration of a PI3K inhibitor together with some HDAC inhibitors compared to the HDAC inhibitor alone in latently infected cells lines and CD4 + T-cells from PWH on ART (Figs 1 and 2).

We propose two potential mechanisms for the enhanced cell death seen with the administration of Pi3K inhibitors and an LRA. The first explanation is that infected cells that expressed HIV RNA (and potentially HIV proteins) were more susceptible to cell death. However, in cell lines following an LRA, death was lower in a latently infected cell line (J-Lat10.6) compared to the uninfected parental cell line (Jurkat). Others have shown that the Tat protein on its own can enhance cell survival of a cell lines [56] which might have contributed to the enhanced survival of the J-Lat10.6 cell line. In contrast to this finding in cell lines, we observed a positive correlation between the fold increase in HIV RNA and reduction in HIV DNA for LRAs alone and LRAs together with PI3K inhibitors (Fig 4). Therefore, another interpretation could be that in the three latently infected cell lines and primary cell experiments there was variation in the potency of latency reversal and this impacted the possible impacts on cell death.

An alternative explanation is that some LRAs can also impact cell death pathways. For example, romidepsin is a bicyclic depsipeptide that inhibits class I HDACs [43]. However, romidepsin can also inhibit the PI3K/Akt pathway in T-cell derived cancer cell lines by reducing the level of pan-Akt and phospho-Akt protein, as well as directly inhibiting the p85 catalytic subunit of PI3K, ultimately leading to the production of reactive oxygen species and an increase in pro-apoptotic molecules [48,57,58]. Therefore, it is possible that romidepsin also enhanced the effects of the PI3K inhibitors through its additional inhibition of the PI3K pathway.

Romidepsin is reported to increase the pro-apoptotic Bak protein and cleavage of caspase-3 in cancer cell lines [48]. Moreover, in other studies with cutaneous T-cell lymphoma cell lines, romidepsin and venetoclax showed a synergistic increase in cell death [59]. In our study using CyTOF, we observed a modest increase in the median expression of Bcl-XL, Bak and cleaved caspase-3 when romidepsin was combined with venetoclax, consistent with romidepsin together with venetoclax largely having an effect on host cell death pathways, rather than through activation of viral transcripts or proteins. Future work, potentially using CITE-Seq (Cellular Indexing of Transcriptomes and Epitomes by sequencing) [60] could provide further insights into the potential mechanism/s of death induced by pro-apoptotic drugs and LRAs within cells simultaneously expressing RNA and pro-apoptotic proteins on the cell surface. We were unable to distinguish between these populations in our CyTOF study. We note that the participant with the greatest change in the pro-apoptotic was on antiretrovirals for 12 years and had a relatively high nadir CD4 count of 538 cells/mm^3^ and a current CD4 count of 864 cells/mm^3^ (S1 Table in S1 File).

Our findings on the impact of some LRAs and venetoclax on the HIV reservoir is consistent with some but not all other prior publications. Using an *in vitro* model of HIV latency, the combination of JQ1 and venetoclax was shown by others to lead to a reduction in integrated HIV DNA [61]. In another study using primary CD4 + T-cells from PWH on ART *ex vivo*, bryostatin and venetoclax reduced total HIV DNA but not replication-competent virus [17]. In contrast to these two prior publications, we found no evidence of synergy between JQ1 or bryostatin and venetoclax in cell lines. There are several explanations for the differences in our findings and previous papers. First, different models of latency were used. For example, Ren et al. examined effects on productively infected cells as they spiked autologous CD4 + T-cells infected with HIV NL4.3 virus into resting CD4 + T-cells from PWH [17], while we used either latently infected cell lines or CD4 + T-cells from PWH on ART *ex vivo*. Second, the concentration of LRAs used in one prior study was 2–3-fold higher than the concentration of romidepsin, JQ1 and bryostatin used in our study with venetoclax [61], and the duration of cultures were also different [28,62].

We demonstrated a reduction in both integrated HIV DNA and intact proviral DNA following venetoclax together with romidepsin, which is important, given that intact HIV DNA can give rise to replication competent virus and defective virus cannot [63]. In contrast and to our surprise, there was little change in cells infected with 5’ and 3’ defective proviruses following incubation with venetoclax and romidepsin (S4 Fig in S1 File). This observation suggests that the depletion of latently infected cells may potentially require production of pro-apoptotic viral proteins not present in defective viruses, such as HIV Envelope protein which would not be produced in 3’ defective viruses [64] or 5‘ defective proviruses [63]. Expression and cytoplasmic export of gag-pol HIV RNA during latency reversal has been shown to activate innate immune signalling [65], which has been linked to inflammatory cell death pathways such as pyroptosis observed in CD4 + T-cells that harbour abortive transcripts [66,67]. This suggests that intact proviruses following activation may be able to trigger additional pathways that drive cell death. Additionally, over time on ART, intact and defective proviruses have been found to integrate into different sites in the DNA genome with intact proviruses integrated in more accessible and transcriptionally active regions [68]. As open chromatin environments support greater inducibility of viral gene expression, cells carrying intact proviruses might be preferentially depleted by romidepsin and venetoclax, as shown in this study*.* Another interpretation is that there could be differences in cell survival pathways in cells that carry intact and defective virus that to date have not been defined, and romidepsin and venetoclax might target cell survival pathways in cells with intact provirus leading to their selective depletion [69,70].

This study of pro-apoptotic drugs and LRAs in latently infected cell lines and cells from PWH on ART *ex vivo* is the first to systematically examine the impact of inhibiting the PI3K pathway on the death of HIV-infected cells. However, this study had some limitations. First, we only quantified integrated and intact HIV DNA and therefore cannot make any conclusions in relation to depletion of cells that contain inducible virus. Another limitation was the study population. In this study, all donors were male. Sex differences are known to be a factor in the efficacy of latency reversal due to immune differences [71]. Given the absence of a viral protein marker in our CyTOF studies, we were unable to distinguish between upregulation of pro- or anti-apoptotic proteins in HIV-infected and uninfected cells. Newer single cell approaches such as FIND-seq [72] and PheP-seq [73] might be helpful in future studies. Additionally, due to the small number of donors in the study, we did not have sufficient power to look at correlations between the decline in integrated HIV DNA and increase in unspliced HIV RNA for each LRA alone. Finally, we were unable to determine if the expression of specific viral proteins enhanced cell death or cell survival, as shown previously with Tat protein [56]. Future work using far more potent LRAs, such as Tat mRNA as we recently reported [74] should be further explored in combination with pro-apoptotic drugs.

## Conclusion

In summary, we combined a range of LRAs with two different classes of drugs that promote cell death, inhibitors of PI3K and inhibitors of B-cell lymphoma-2, and tested the effects on the HIV reservoir in latently infected cell lines and cells from PWH on ART. In the latently infected cell line J-Lat10.6 and in the uninfected Jurkat cell line, we observed increased cell death following an LRA together with either inhibitors of PI3K or venetoclax, compared to an LRA alone. Using cells from PWH on ART, both wortmannin and venetoclax reduced the HIV reservoir when used alone. However, there was no significant differences in the decline of HIV DNA when we compared LRA alone to LRAs with PI3K inhibitors. Similarly, there was no difference in the decline of HIV DNA or intact proviral DNA when cells were treated with venetoclax alone compared to a combination of venetoclax with romidepsin. Overall, these results highlight minimal additional efficacy in reservoir reduction when combining currently available LRAs and pro-apoptotic drugs.

## Supporting information

S1 File S1-S6 Figs, S1 and S2 Tables. (DOCX)

S1 Raw Data (XLSX)
