# Supplementary material for: The effect of combining HIV latency reversal with inhibition of phosphoinositide-3 kinases or B-cell lymphoma-2 on the HIV reservoir
Source: PLoS Pathog. 2026 Jan 29;22(1):e1013923. doi: 10.1371/journal.ppat.1013923 (PMC12880745; doi:10.1371/journal.ppat.1013923)
Supplement: S1 File — (DOCX) [file ppat.1013923.s001.docx]

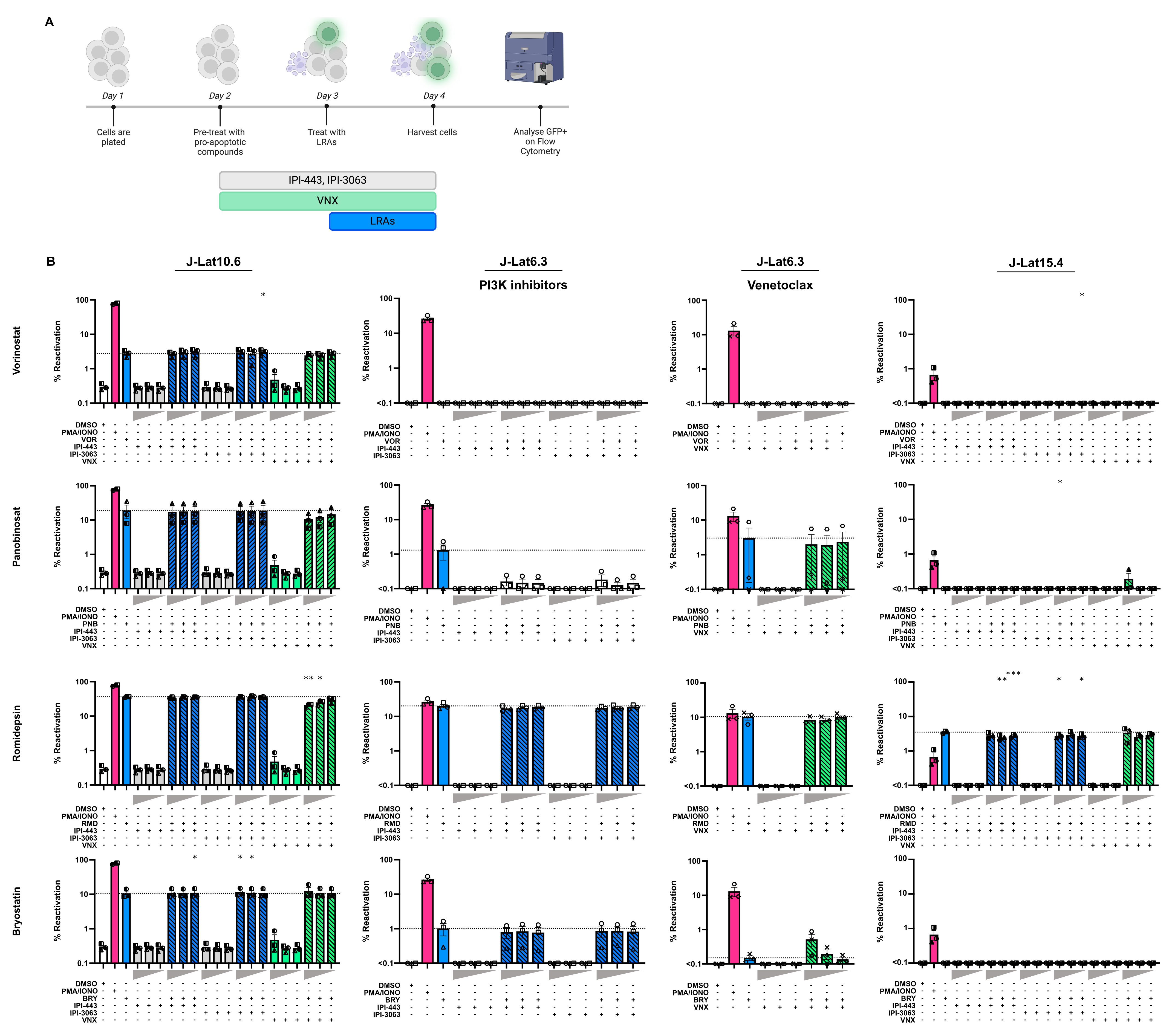


**Supplementary Figure 1. Latency reversal in latently infected T-cell lines following incubation with LRA and pro-apoptotic drugs.**

(A) Schematic of the experimental plan. J-Lat10.6, J-Lat6.3 or J-Lat15.4 T-cell clones were pre-treated with decreasing concentrations of the PI3K inhibitors IPI-443 or IPI-3063 (100, 10 or 1 nM) or Venetoclax (1000, 100 or 5 nM) for 24 hours. The latency reversing agents (LRAs): vorinostat (VOR; 1000nM), romidepsin (RMD; 40nM) panobinostat (PNB; 30nM), bryostatin (BRY; 12.5nM), or PMA/Ionomycin (PMA/IONO; 16nM and 0.5uM) as a positive control, was then added to cell cultures for an additional 24 hours. Cells were harvested 24 hours after the addition of LRAs, stained with a Live/Dead Fixable Violet Dead Cell Stain, and analysed using flow cytometry. (B) The percentage of reactivated cells expressing GFP following stimulation with either: PMA/IONO as a positive control (pink), each LRA alone (blue), PI3K inhibitor alone (light grey) or in combination with each LRA (blue lined), Bcl-2 inhibitor alone (green) or combined with each LRA (green lined), is shown. Reactivation of J-Lat cell clones 10.6 (far left column), 6.3 (middle columns) and 15.4 (far right column) is shown. Each symbol represents the average of technical replicates and represents an independent experiment. Height of the column is the mean and the error bars, SEM. The horizontal dashed line indicates the percentage of reactivated cells following treatment of J-Lat cells with the specific LRA alone. A paired t-test on raw values was used to calculate statistical significance, *p<0.05, **p<0.01, ***p<0.001. Created in BioRender. Kim, Y. (2026) <https://BioRender.com/o9vg71a>


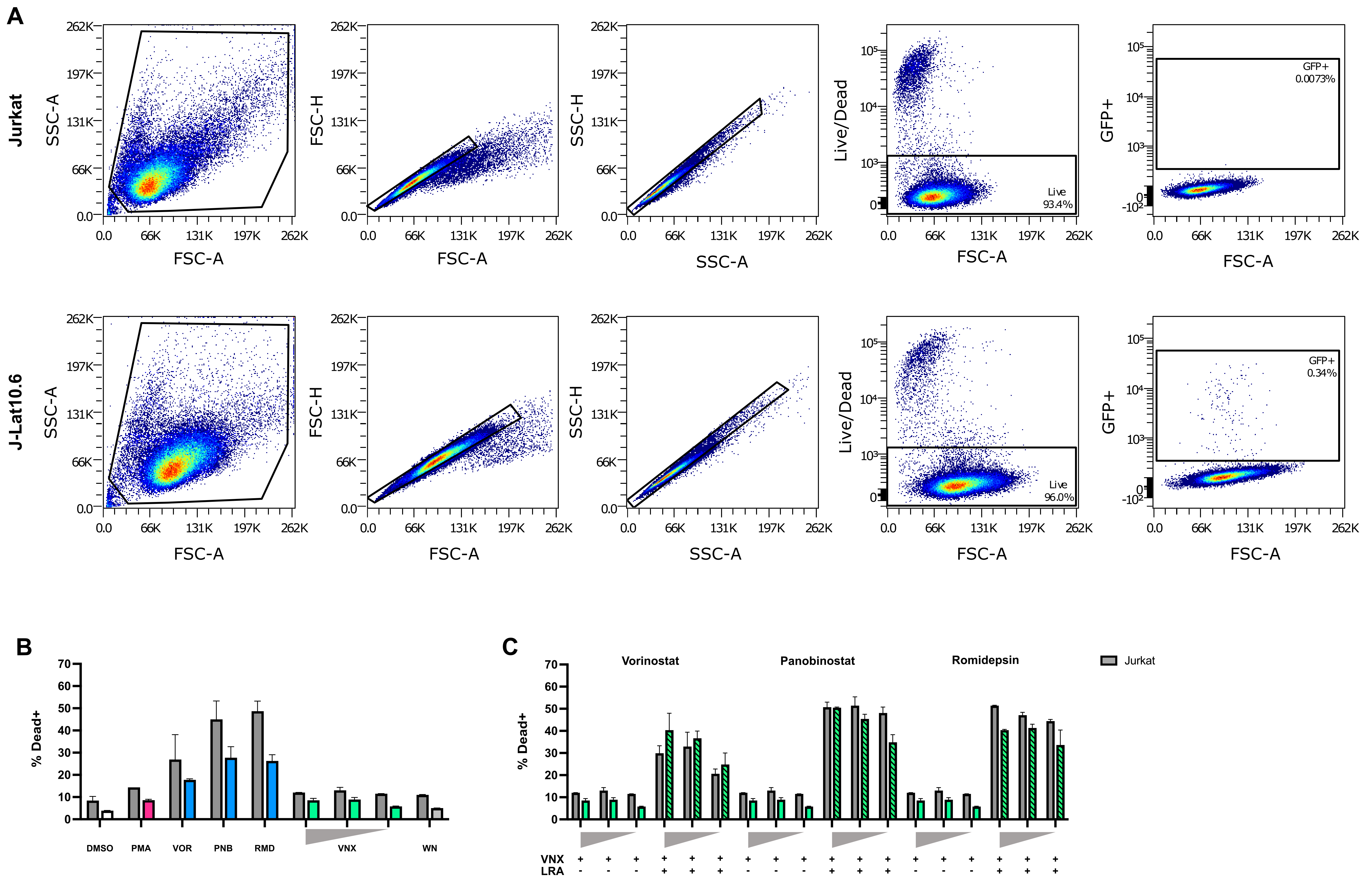


**Supplementary Figure 2.** **Toxicity of LRAs and PI3K inhibitors in Jurkat and J-Lat10.6 T-cells.**

(A) Flow cytometry and gating strategy for Jurkat (top row) and J-Lat10.6 T-cell lines (bottom row). Cell lines were gated on forward and side scatter, followed by two different gates to eliminate doublet cells. Cell viability was determined using a Live/Dead Fixable Cell death stain. We then gated on live cells and determine the percentage that expressed green fluorescent protein (GFP). (B) The percentage of dead cells using a live/dead stain are shown for Jurkat (dark grey bars) and J-Lat10.6 (coloured bars) T-cells following treatment for 24 hours with the LRAs vorinostat, panobinostat and romidepsin (blue bars) or the pro-apoptotic drugs wortmannin (light grey bars) or venetoclax (green bars; at reducing concentrations i.e. 100, 10 and 5 nM, indicated by a triangle) or (C) following pre-incubation with venetoclax (green bars; at reducing concentrations i.e. 100, 10 and 5 nM, indicated by a triangle) for 24 hours followed by the addition of either vorinostat, panobinostat or romidepsin (green hashed bars). Height of the column is the mean of two independent experiments and the error bars, SEM.


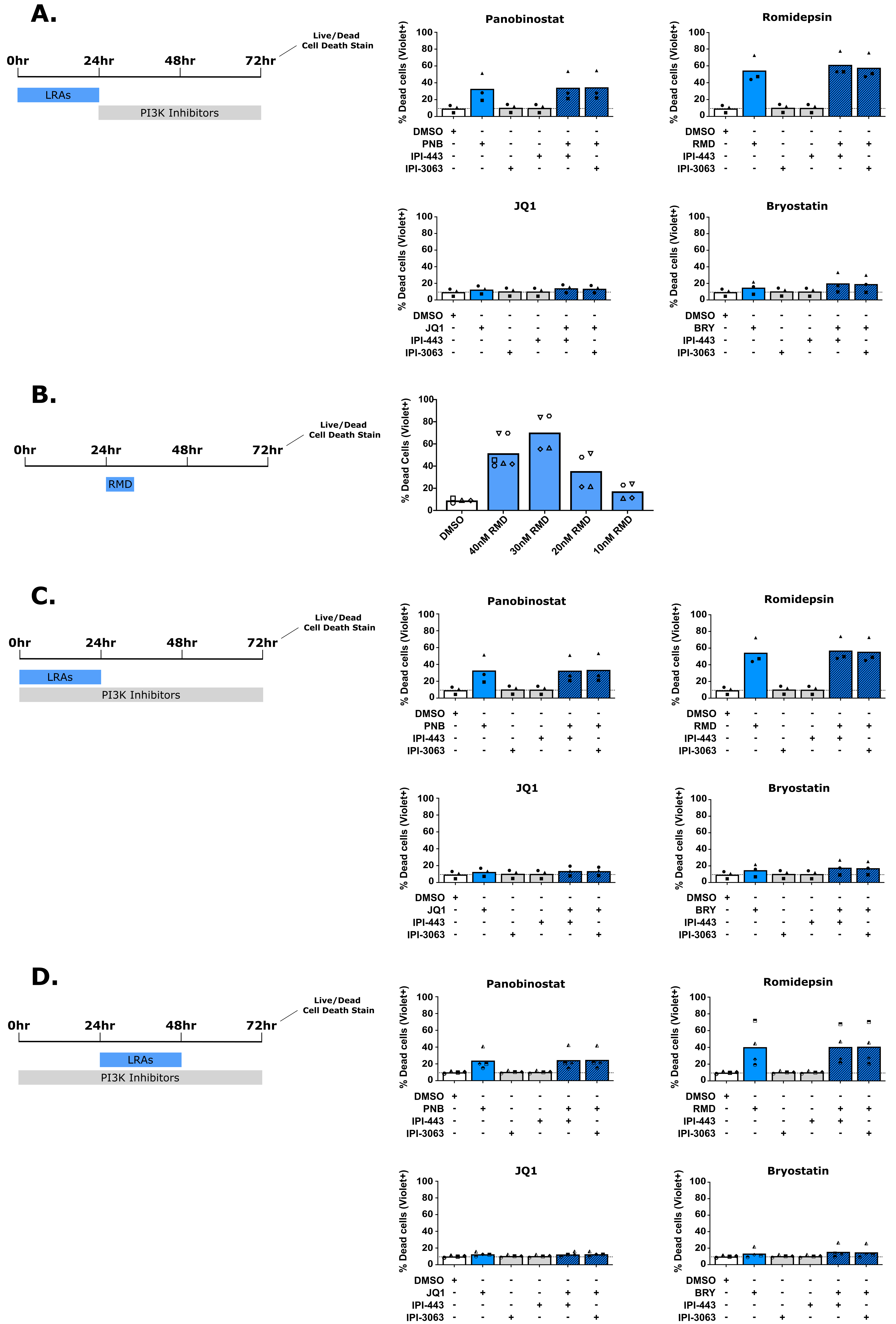


**Supplementary Figure 3.** **Toxicity of LRAs and PI3K inhibitors in primary CD4+ T-cells treated with different dosing strategies.**

Resting CD4+ T-cells were isolated from uninfected PBMCs from healthy donors. Cells were treated with PI3K inhibitors and latency reversing agents (LRAs) using different dosing strategies: A) 24 hour pulse with LRA alone followed by 48 hours with PI3K inhibitors alone; B) 4 hour pulse with varying concentrations of romidepsin, then cultured for additional 48 hours, C) 24 hour pulse of LRAs together with PI3K inhibitors, followed by 48 hours of PI3K inhibitors alone; and D) pre-treatment with PI3K inhibitors alone for 24 hours followed by a 24 hour pulse of LRA plus PI3K inhibitors, followed by PI3K inhibitors alone for 24 hours. 72 hours after culture, all cells were stained with the Live/Dead Fixable Violet Dead Cell stain and analysed by flow cytometry for the percentage of dead cells. All cells were stained with the Live/Dead Fixable Violet Dead Cell stain and analysed by flow cytometry to determine the percentage of dead cells. PNB-30nM panobinostat; RMD- 40nM romidepsin (unless another concentration is written); JQ1 (1000nM); BRY- 12.5nM bryostatin. Each symbol denotes a different donor, n≤6. The height of the column represents the mean, and error bars the SEM. Each symbol is a different donor and the average of technical replicates.


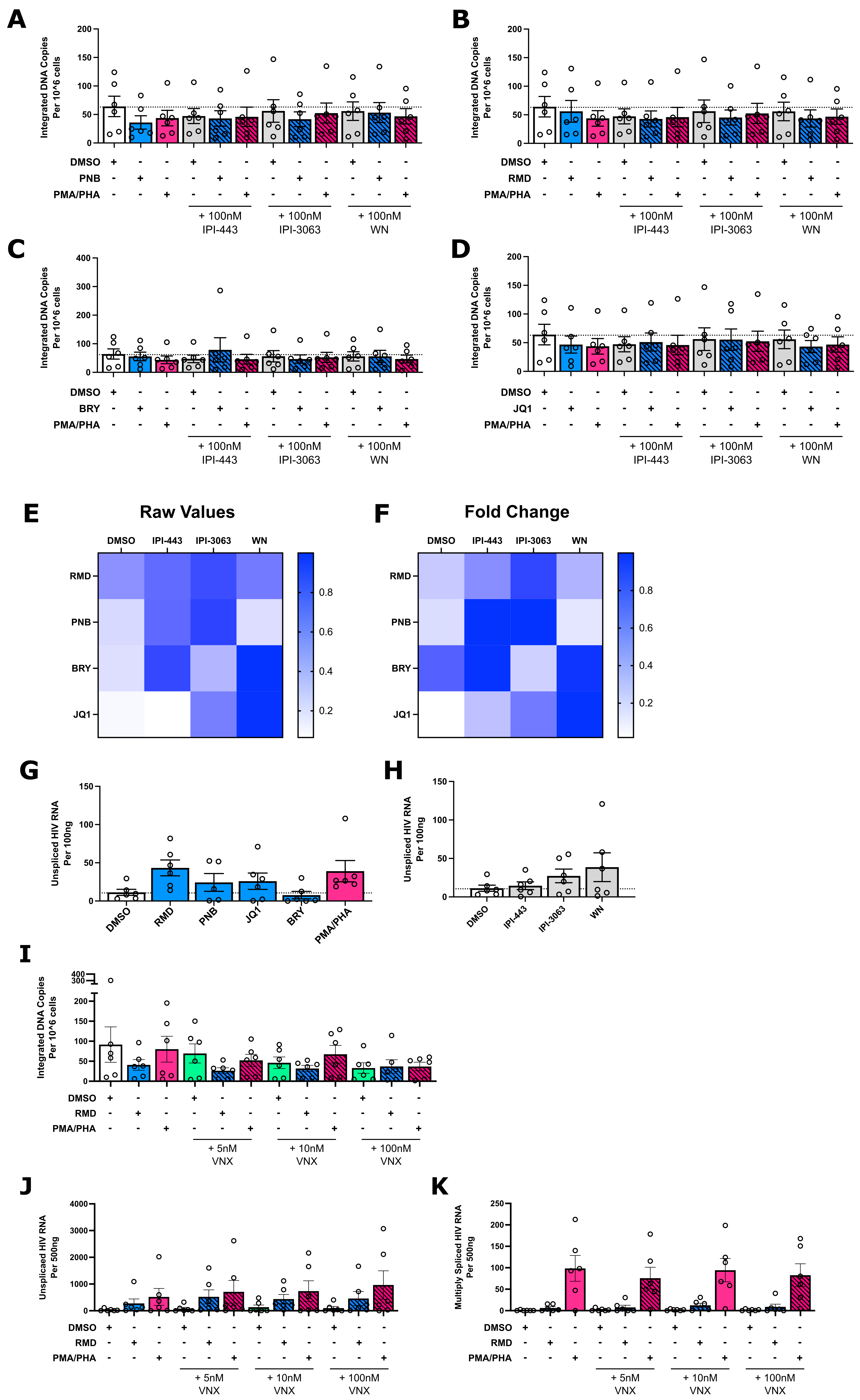


**Supplementary Figure 4. Levels of integrated HIV DNA and cell associated HIV RNA following treatment with LRAs combined with pro-apoptotic drugs.**

Total CD4+ T-cells isolated from PWH on ART were pre-treated with pro-apoptotic drugs [100nM IPI-443, 100nM IPI-3063 or 100nM wortmannin (WN)], or DMSO for 24 hours and were then treated with either DMSO or LRAs for 24-hours [panobinostat 30nM (A); romidepsin 20nM (B); bryostatin 12.5nM (C); or JQ1 1000nM (D)], except for romidepsin which was pulsed for 4 hours. All samples were harvested and analysed for HIV integrated DNA and cell associated HIV RNA using qPCR. Differences between conditions in the levels of integrated HIV DNA for (E) raw values and (F) fold-change over DMSO are represented as a heatmap. Raw values for unspliced HIV RNA are shown for cells treated with (G) LRAs alone or (H) LRAs with pro-apoptotic drugs. Total CD4+ T-cells were treated with venetoclax (5nM, 10nM or 100nM) for 24hours prior to the addition of 20nM romidepsin for 4-hours. Cells were harvested and DNA and RNA quantified by qPCR. The absolute values for HIV integrated DNA (I), unspliced HIV RNA (J) and multiply spliced HIV RNA (K) are shown. For all graphs, the height of the column and error bars represent the means and standard error of mean (SEM). Each symbol depicts a different donor.


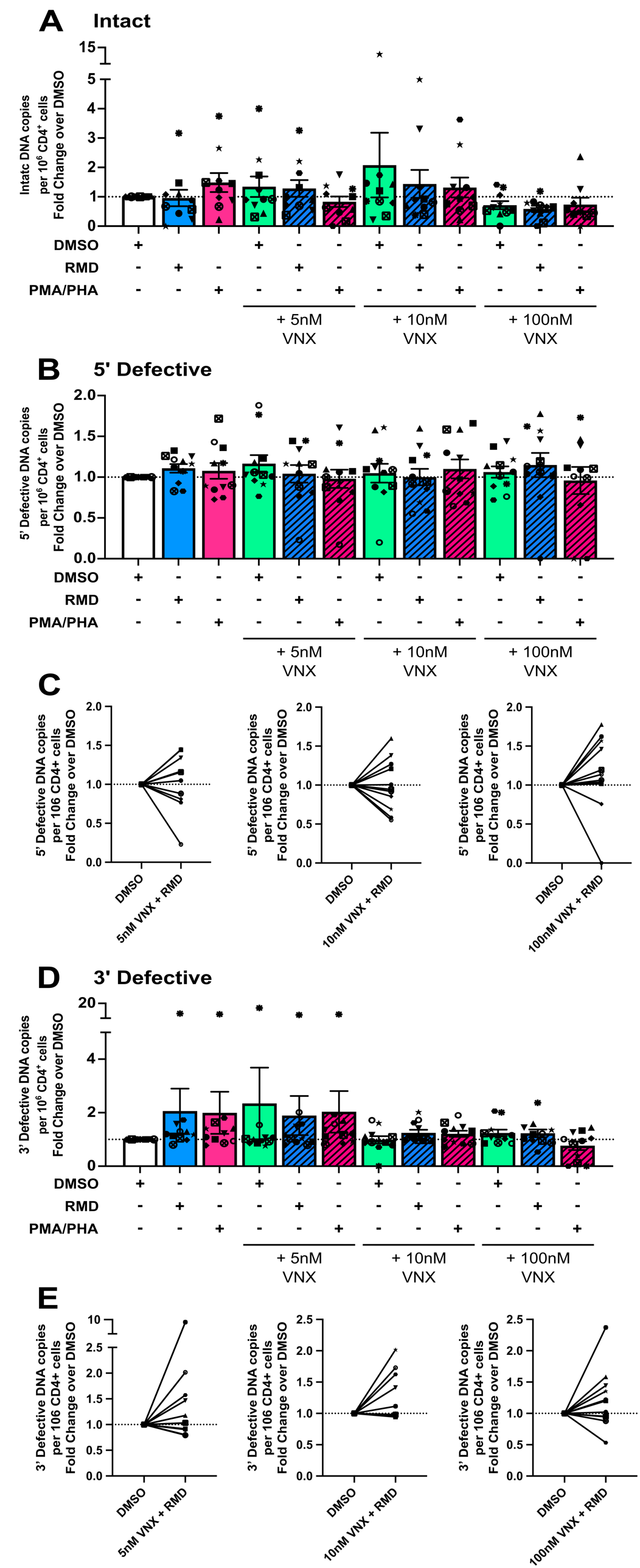


**Supplementary Figure 5. No significant change in 5’ and 3’ defective virus was observed in CD4+ T-cells from PWH on ART *ex vivo* treated with venetoclax and romidepsin.**

CD4+ T-cells from PWH on ART *ex vivo* were treated with 20nMromidepsin and venetoclax (5, 10, 100nM) doses. Genomic DNA was extracted from cells and levels of 5’ defective (A, B) and 3’ defective (C, D) proviral DNA was measured using the Intact Proviral DNA Assay (IPDA). The fold change relative to DMSO is shown for intact (A), 5’ defective (B) and 3’ defective (D) proviral DNA. The change in each specific donor is shown for 5’ defective (C) and 3’ defective (E) proviral DNA. p-values were calculated using a paired t-test on fold change data compared to DMSO. All p values were above 0.05 and therefore differences were not statistically significant. Each symbol represents a different donor. The height of the column and error bars represents the mean and standard error of mean (SEM).


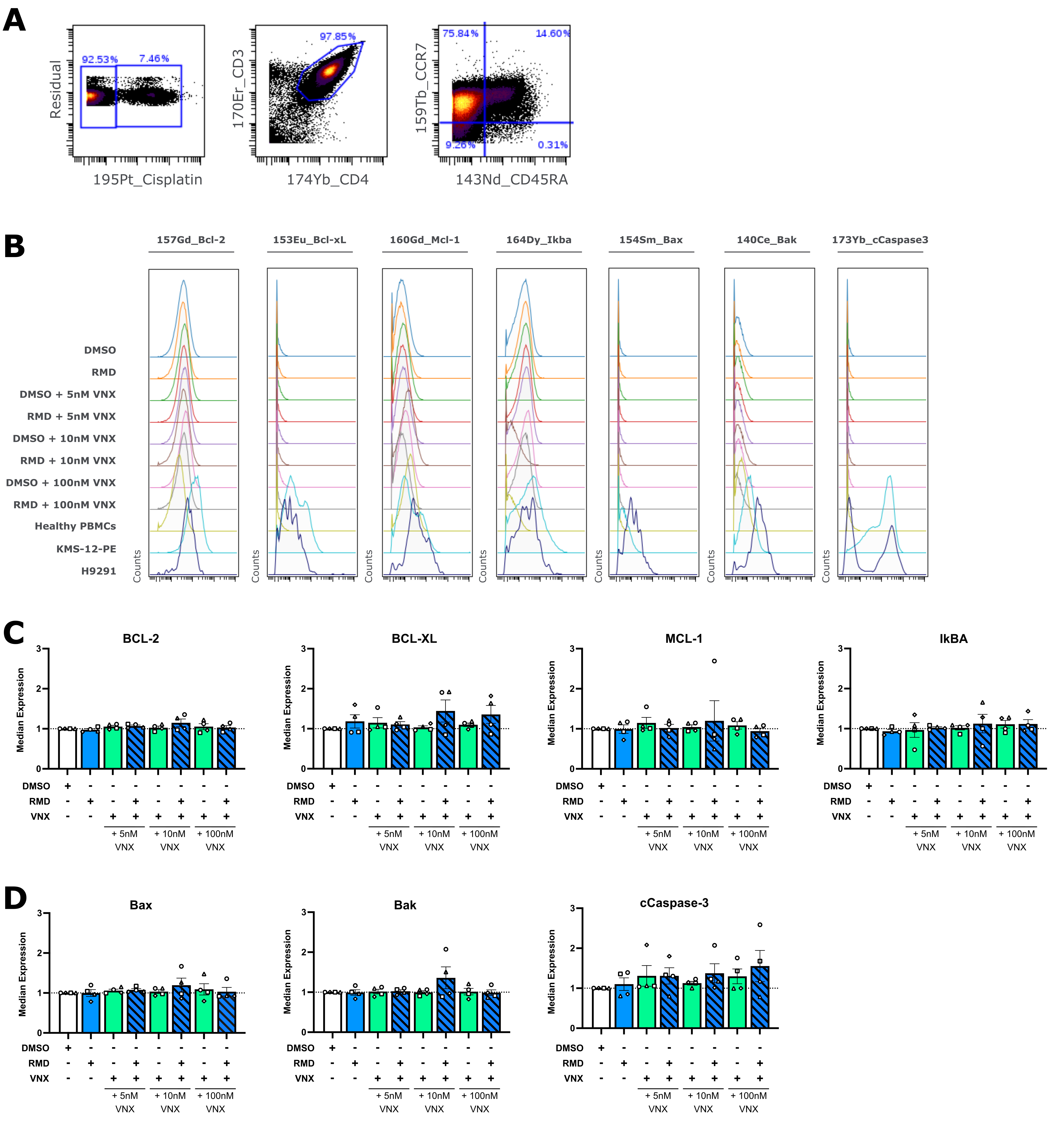


**Supplementary Figure 6. Impact of romidepsin and venetoclax on the differential expression of pro- and anti-apoptotic proteins in CD4+ T-cells from PWH on ART *ex vivo***.

CD4+ T-cells from PWH on *ART ex vivo* were isolated, treated with 20nM romidepsin for 4 hours and 5, 10 or 100nM venetoclax for 24 hours. All drugs were washed out and cells cultured for an additional 24 hours. Cells were washed and stained with cisplatin to discriminate dead cells, and a panel of antibodies conjugated to different metal isotypes that bind to different cellular markers of apoptosis and T-cell subset differentiation. Samples were analysed using cytometry time-of-flight (CyTOF). (A) Flow cytometry plot demonstrating the gating strategy for detection of live and dead cells using cisplatin, followed by quantification of different CD4+ T-cell subsets. (B) Expression of anti- and pro-apoptotic markers from a single donor in the presence of romidepsin and/or different doses of venetoclax, or DMSO. Median expression of pro-apoptotic (C) and anti-apoptotic (D) proteins for CD4+ T-cells following treatment with venetoclax and romidepsin (n=4). Each symbol represents a different donor. Columns represent the mean with error bars denoting the SEM.


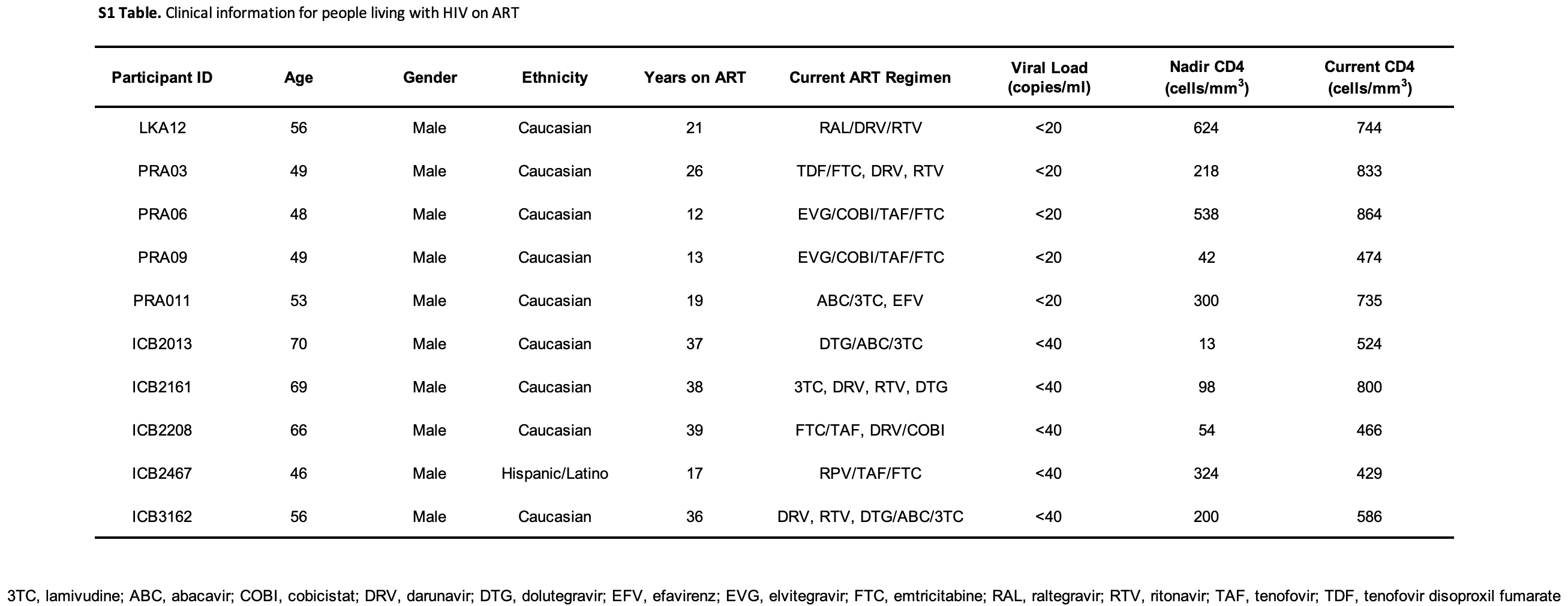


S2 Table. Pro-apoptotic and LRA drug compounds and their mechanism of action

| **Drug Type** | **Drug Compound** | **Mechanism of Action** |
| --- | --- | --- |
| Pro-apoptotic | IPI-443 | Potent dual inhibitor of PI3Kδ/γ isoforms^[1]^ |
|  | IPI-3063 | Potent PI3Kδ isoform inhibitor^[1]^ |
|  | Wortmannin | Non-specific pan-PI3K inhibitor^[2]^ |
|  | Venetoclax | Inhibitor of anti-apoptotic molecule Bcl-2^[3, 4]^ |
| LRA  HDACi | Vorinostat,  Romidepsin  Panobinostat | Remodel chromatin structure to reactivate  latent HIV, displace histone acetylase^[5]^ |
| Protein Kinase C (PKC) agonist | Bryostatin | Stimulate the PKC pathway leading to the recruitment of NF-κB to reactivate latent HIV^[6]^ |
| Bromodomain Inhibitor | JQ1 | Dissociates bromodomain-4 from the HIV protomer, allowing stimulation of HIV elongation^[7, 8]^ |

References

1. Chiu H, Mallya S, Nguyen P, Mai A, Jackson LV, Winkler DG, et al. The Selective Phosphoinoside-3-Kinase p110delta Inhibitor IPI-3063 Potently Suppresses B Cell Survival, Proliferation, and Differentiation. Front Immunol. 2017;8:747. doi: 10.3389/fimmu.2017.00747. PubMed PMID: 28713374; PubMed Central PMCID: PMCPMC5491903.

2. Powis G, Bonjouklian R, Berggren MM, Gallegos A, Abraham R, Ashendel C, et al. Wortmannin, a potent and selective inhibitor of phosphatidylinositol-3-kinase. Cancer Res. 1994;54(9):2419-23. PubMed PMID: 8162590.

3. Cummins NW, Sainski AM, Dai H, Natesampillai S, Pang YP, Bren GD, et al. Prime, Shock, and Kill: Priming CD4 T Cells from HIV Patients with a BCL-2 Antagonist before HIV Reactivation Reduces HIV Reservoir Size. Journal of virology. 2016;90(8):4032-48. doi: 10.1128/JVI.03179-15. PubMed PMID: 26842479; PubMed Central PMCID: PMCPMC4810548.

4. Arandjelovic P, Kim Y, Cooney JP, Preston SP, Doerflinger M, McMahon JH, et al. Venetoclax, alone and in combination with the BH3 mimetic S63845, depletes HIV-1 latently infected cells and delays rebound in humanized mice. Cell Rep Med. 2023:101178. Epub 20230823. doi: 10.1016/j.xcrm.2023.101178. PubMed PMID: 37652018.

5. Rasmussen TA, Tolstrup M, Brinkmann CR, Olesen R, Erikstrup C, Solomon A, et al. Panobinostat, a histone deacetylase inhibitor, for latent-virus reactivation in HIV-infected patients on suppressive antiretroviral therapy: a phase 1/2, single group, clinical trial. The Lancet HIV. 2014;1(1):e13-e21. doi: 10.1016/s2352-3018(14)70014-1.

6. Gutierrez C, Serrano-Villar S, Madrid-Elena N, Perez-Elias MJ, Martin ME, Barbas C, et al. Bryostatin-1 for latent virus reactivation in HIV-infected patients on antiretroviral therapy. AIDS. 2016;30(9):1385-92. doi: 10.1097/QAD.0000000000001064. PubMed PMID: 26891037.

7. Li Z, Guo J, Wu Y, Zhou Q. The BET bromodomain inhibitor JQ1 activates HIV latency through antagonizing Brd4 inhibition of Tat-transactivation. Nucleic acids research. 2013;41(1):277-87. doi: 10.1093/nar/gks976. PubMed PMID: 23087374; PubMed Central PMCID: PMCPMC3592394.

8. Boehm D, Calvanese V, Dar RD, Xing S, Schroeder S, Martins L, et al. BET bromodomain-targeting compounds reactivate HIV from latency via a Tat-independent mechanism. Cell cycle. 2013;12(3):452-62. doi: 10.4161/cc.23309. PubMed PMID: 23255218; PubMed Central PMCID: PMCPMC3587446.
